# Editorial: Clinical manifestations and comorbidities in axial and peripheral spondyloarthritis

**DOI:** 10.3389/fmed.2022.980732

**Published:** 2022-08-12

**Authors:** Ruxandra Elena Schiotis, Eduardo Collantes-Estévez, Clementina López-Medina

**Affiliations:** ^1^Department of Rheumatology, Clinical Hospital of Infectious Diseases, Cluj-Napoca, Romania; ^2^Department of Rheumatology Reina Sofia University Hospital, Maimonides Biomedical Research Institute of Cordoba (IMIBIC), University of Cordoba, Cordoba, Spain

**Keywords:** axial spondyloarthritis, peripheral spondyloarthritis, comorbidities, clinical manifestations, treatment

In recent years, important advances in the understanding of the clinical picture of SpA have been made. However, the natural evolution of the disease, the appearance of extra-articular manifestations, the development of comorbidities and the impact of such manifestations on treatment management and outcomes should be studied in depth. Some of these critical aspects are discussed in the Research Topic entitled: Clinical manifestations and comorbidities in spondyloarthritis (SpA).

This Research Topic gathers seven papers from different research groups around the world that deal with distinct aspects of clinical manifestations and comorbidities in SpA. Five of them are original research, one is a systematic review and another one is a clinical perspective.

One of the many extra-articular manifestations in SpA is the aortic valve involvement. Tissue degeneration near the atrioventricular node by inflammation of the aortic valve attachment site can lead to electrical conduction disorders expressed in the form of atrioventricular block (AVB) or branch block (BBB). In their systematic review, Park et al. assessed the prevalence and risk for these electric disorders and also for the pacemaker implantation (PMI) in patients with SpA. The main findings revealed that although there were no significant differences in prevalence of low grade AVB and BBB in SpA patients, a double-fold increased risk of high grade AVB and PMI was identified in all types of SpA.

A very frequent extra-articular manifestation in SpA involves the gut, reaching a prevalence of 6–14% in case of clinically expressed intestinal inflammation, while the incidence of subclinical gut inflammation is up to 44–60% among patients with SpA (1). Consequently, it is necessary to identify an effective screening tool which can be able to detect a suspected intestinal inflammation among patients with SpA. In their paper, the authors Feng et al. identified that the value of D-dimer was increased in patients with SpA compared with healthy controls and was positively correlated with C reactive protein, erythrocyte sedimentation rate and thrombocyte level. The study further mentions that the raised D-dimer value is related to peripheral joint involvement and gut inflammation.

Obesity is a prevailing comorbidity in several inflammatory rheumatic diseases (2), with particular relevance in psoriatic arthritis (PsA) patients (3). It is known already that anti-TNF agents may affect the weight and body composition of treated patients (4), in turn, obesity may influence the clinical response to anti-TNF agents (5). In relation with this García-Dorta et al. identified in their study that obesity was not associated with a lower secukinumab retention rate. In addition, in this multicenter real-world study of the retention rate of secukinumab authors identified that the patient groups with a better secukinumab retention rate were women with axial SpA and men with PsA.

Furthermore, Zhao et al. reminded us that osteomalacia can mimic the clinical and even radiological presentations of SpA. The study of Zhao et al. showed that there were many similarities in the clinical presentations of patients with osteomalacia and SpA, but generalized pain and myasthenia was only developed by osteomalacia patients. Concerning radiological abnormalities, one of the leading causes of osteomalacia being misdiagnosed as SpA were the sacro-iliac joints MRI abnormalities.

In order to point out that SpA treatment may also induce (co)-morbidity Queiro-Silva et al. in their perspective article on non-steroidal anti-inflammatory drugs (NSAIDs) offer a new image on how NSAIDs could restrain the goals of pharmacological treatment at medium and long-term in SpA. Apart from their short-term symptom control role, the authors invite us to reconsider the place of NSAIDs in the disease management since NSAIDs are responsible for 30% of hospitalizations for preventable adverse drug reactions (6). NSAIDs can also determine changes in the composition of the intestinal microbiota, promoting an alteration of the barrier function in the intestinal epithelium. This event may further activate the type-3 immunity in the intestinal lamina propria, as the authors very nicely pointed out.

On the other hand, Guo et al. present an exciting approach to assess disease activity in SpA by prospectively looking into the relationship between intravoxel incoherent motion (IVIM) diffusion-weighted imaging (DWI) and dynamic contrast-enhanced MRI (DCE-MRI) parameters of sacroiliitis. The results of their study showed that for measuring the disease activity, quantitative parameters were superior to semi-quantitative parameters from DCE-MRI. Authors also established a correlation between quantitative parameters from IVIM DWI and DCE-MRI in axSpA.

Not least, D'Angelo et al. presents The “Early SpA Clinic” project that aims to help Rheumatology Centers in establishing specific organizational solutions to solve problems related to early diagnosis and follow-up of patients with SpA. The results accomplished by their project are promising, particularly in terms of better management of resources, personnel, spaces and equipment related to the proportions of patients.

In conclusion, these articles show on one hand that significant advances in the knowledge of clinical manifestations and comorbidities of SpA have been made but there is still a need for more research in this field in order to better define these heterogeneous group of diseases.

## Author contributions

All authors listed have made a substantial, direct, and intellectual contribution to the work and approved it for publication.

## Conflict of interest

The authors declare that the research was conducted in the absence of any commercial or financial relationships that could be construed as a potential conflict of interest.

## Publisher's note

All claims expressed in this article are solely those of the authors and do not necessarily represent those of their affiliated organizations, or those of the publisher, the editors and the reviewers. Any product that may be evaluated in this article, or claim that may be made by its manufacturer, is not guaranteed or endorsed by the publisher.
